# Perceiving Oneself to Be Integrated into the Peer Group: A Protective Factor against Victimization in Children with Learning Disabilities

**DOI:** 10.3390/brainsci13020263

**Published:** 2023-02-03

**Authors:** Mara Marini, Gloria Di Filippo, Marika Bonuomo, Giulia Torregiani, Stefano Livi

**Affiliations:** 1 Department of Social and Developmental Psychology, Sapienza University of Rome, 00185 Rome, Italy; 2 Faculty of Psychology, Niccolò Cusano University, 00166 Rome, Italy

**Keywords:** school bullying, social integration, specific learning disabilities

## Abstract

Bullying is still a widespread social problem that needs serious attention. To date, research on this topic has shown that understanding the phenomenon requires a psychosocial perspective. The primary goal of the study is to identify the factors that contribute to the victimization of students with learning disabilities. The hypothesis is that the victimization experiences of this group of students can be explained by some socio-relational dynamics. Using a mediation model, this study demonstrates that perceived social integration completely mediates the association between the presence of learning disabilities and victimization experiences. This implies that students with learning disabilities are primarily victimized when they are not socially integrated into their class group. The implications for diagnosis and treatment are discussed.

## 1. Introduction

Long-standing research on children with Specific Learning Disabilities (SLDs) has focused on academic adaptability and the cognitive processes engaged in, and thus impeding, learning processes—as well as intervention strategies that can be successfully deployed to assist these students in coping with school issues, e.g., [1,2,3,4,5,6]. In contrast, despite their importance in the school context, e.g., [7,8,9,10], socio-affective dynamics have been partially overlooked when it comes to children and adolescents with learning disabilities [11]. However, it is now recognized that students’ academic performance and achievements–especially when compared to those of their peers [12,13]—influence various school-related experiences as well as how children and adolescents perceive themselves, e.g., [14,15,16]. Cognitive problems, which impede academic achievement in students with learning disabilities, are risk factors for psychosocial disorders, further limiting their ability to learn.

Many studies in the literature have been interested in the bullying phenomenon within the complex framework of learning disabilities, beginning with the idea that the emotional and relational profiles of students with SLDs share many similarities with bullies and victims. For instance, bullies have distinctive characteristics such as poor academic performance, emotional distress, suicidal and self-harming thoughts, and low self-esteem, all of which can be found in children and adolescents with SLDs [17,18,19,20]. In addition to academic difficulties, students with learning disabilities (compared to typically developed students) are more likely to develop internalizing and/or externalizing disorders, e.g., [21,22,23,24], and have limited or inadequate social skills, e.g., [25,26,27,28,29,30,31], that prevent them from positively interacting with peers. As a result, students with learning difficulties present a number of social problems [32]. Reading difficulties, for example, have been linked to peer rejection [32]. Indeed, students with SLDs appear to be more socially isolated and less accepted by their peers [33,34,35]. It has been demonstrated in this regard that learning disabilities are reflected not only in the setting of academic achievements but also in the socio-affective dynamics of students [30]. Compared to neurotypical students, students with learning disabilities have fewer friends, higher rates of social isolation, decentralized positions in social networks, and spend most of their time with peers who share their problematic social traits, e.g., [36]. According to studies in this field, these characteristics make students with learning disabilities more susceptible to aggressive behavior in the classroom [37,38,39,40].

Several studies have attempted to shed light on the relationship between SLDs and victimization vulnerability. In particular, Mishna [41] used the concept of double jeopardy to explain the increased vulnerability of students with learning disabilities in bullying studies. Based on this perspective, students with learning disabilities are more vulnerable to victimization because of their reduced ability to interpret social cues and respond appropriately, resulting in additional emotional and psychological difficulties. Indeed, the so-called “snowball effect” [41] suggests that such emotional and psychological issues may lead to a decline in academic performance, and the increased visibility of learning difficulties may expose these students to a higher risk of victimization, e.g., [40,42,43,44,45,46,47,48].

Despite a growing body of literature examining the dynamics of bullying among students with disabilities, fewer studies have been conducted than on students without disabilities [42,47,49]. In light of our societies’ focus on the inclusion and integration of vulnerable students, this constitutes a limitation of the available literature. Indeed, a number of researchers have documented the overrepresentation of students with disabilities in school bullying data and demonstrated that bullying rates could be influenced by the type of disability [45,46,48]. However, some studies have produced inconsistent results, e.g., [47,50]. Probably, several factors can explain these discrepancies, including the ambiguous definition of bullying, methodological issues, demographic characteristics (e.g., age, gender, race, or ethnicity), and social contextual or cultural factors in which bullying occurs, e.g., [46]. Specifically, as previously described, the overrepresentation of students with learning disabilities in school bullying was frequently attributed to their characteristics, while social dynamics in the classroom were largely disregarded [51,52]. In addition, research on bullying from a social-ecological perspective [53] suggests that this phenomenon can be better understood by focusing on interactions between the individual and their social environments [54]—including their family, peer group, educational community, and society. This perspective emphasizes the need to understand bullying from a psychosocial standpoint [47], going beyond the individual vulnerability elements.

Recent studies point in this direction. In recent years, there has been an increased focus on sophisticated forms of aggressive behavior among peers—also known as discriminatory bullying [55]—in which victimization is a result of minority group membership (for age, sexual orientation, gender identity, religion, disability, and ethnicity) and is thus motivated by individual and/or cultural prejudices [56]. Indeed, a person’s social affiliations and identities derived from them serve as an indispensable point of reference. According to the Theory of Social Identity [57], the need to belong and positive social identification, in conjunction with social comparison mechanisms, can explain aggressive behavior toward minority outgroups. In this context, students with disabilities are more likely to be victimized by their peers because they may be perceived as “different” [45]. These social categorization processes can aid researchers in understanding the motivations underlying the aggressive behavior of bullies toward students from diverse minority groups. Focusing on how students perceive themselves in relation to their reference groups, such as the class group, the psychosocial approach from the victim’s perspective may provide a new lens through which to examine the issue. 

The efficacy of prevention and intervention strategies that target individuals and their social environment, particularly within the classroom, demonstrates the significance of a psychosocial perspective in the study of bullying [58]. These studies have demonstrated that a positive school climate is associated with lower bullying rates [59] and greater social competence, e.g., [60,61]. Positive and inclusive school environments foster stronger peer relationships and shield vulnerable students from victimization, e.g., [44]. Classes are places where all students can fulfill their psychological needs [62,63], and the peer group is essential for developing a sense of belonging. It implies that the social context is important for all students, but it is crucial for those with disabilities.

Following the preceding explanations, the current study explored school bullying by considering the socio-relational variables that may be involved in the phenomenon. Although the evidence suggests that a disability increases the risk of victimization, the prevarications to the detriment of students with learning disabilities, when present, cannot be examined without reference to the socio-contextual factors that define the environment in which the abuse occurs. Therefore, the purpose of the proposed study is to highlight the disadvantages of studying SLDs as clinical entities that can be rapidly identified and nosographically defined. When considering the phenomenon of bullying against this group of students, we have chosen to focus on the classroom sense of social integration as a possible victimization-related factor. Taking into consideration aforementioned scientific findings, the primary objective of this contribution is to give voice to the feelings of diversity and self-perception that may characterize the school experience of all students and distinguish the school experience of students with SLDs. It should be noted that children and adolescents with SLDs may experience feelings of rejection and engage in socially ineffective behaviors toward peers. Specifically on this point, the scientific literature reveals inconsistencies regarding the relationship between learning disabilities and bullying.

Assuming that learning difficulties have a significant impact on the interpersonal relationships, our study sought to comprehend the factors that can explain, at least in part, the phenomenon of focusing on the school environment as a site of perpetration of violence against diversity, e.g., [64]. Specifically, we considered the perception of social integration in the classroom as a variable capable of illuminating the possible relationship between learning difficulties and victimization.

**Figure 1:**
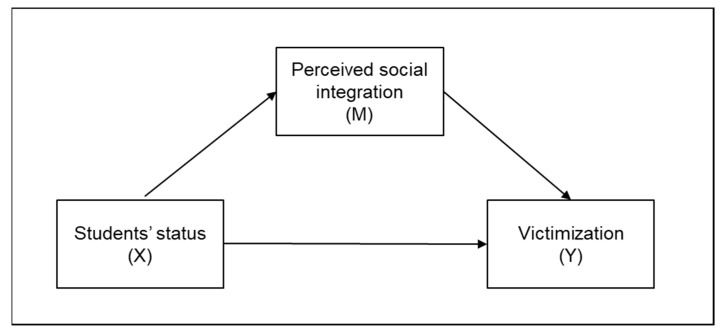
Research model.

For the reasons mentioned above, we hypothesized that students with learning disabilities would be more vulnerable to victimization than those without learning disabilities (Hypothesis 1). We were also interested in determining if children with learning disabilities perceived themselves as less socially integrated into group-class settings (Hypothesis 2). Further, we hypothesized that self-perception of social integration would act as a mediator to explain the association between learning disabilities and peer victimization (Hypothesis 3). The following model summarizes the expectations (Figure 1).

## 2. Materials and Methods

### 2.1. Data Collection and Sample Features

The research was carried out in an Italian lower secondary school. We offered to the school that all students be screened for specific learning difficulties and a study into the impact of peer interactions on psychosocial adjustment. Following the Headmaster’s approval of the study, the research team provided the school with informed consent forms for the students’ families, as a necessary precondition for starting the research activities. Only students whose parents supplied the signed informed consent form were included in the study. The preliminary group consisted of ninety-eight students (60.2% male). Specifically, at the time of compilation, there were twenty-six students in the first class, sixty-five students in the second class, and seven students in the third class. The screening for specific learning difficulties and questionnaire completion occurred during school hours. Participants were led into a classroom supplied by the school and performed all tests with the assistance of the researchers. The study was conducted in accordance with the Declaration of Helsinki and with the approval of the University Ethics Committee. In the initial stage, each participant received screening to determine whether or not they had an SLD. Student groups with SLDs and those without SLDs were created using this approach, which divided the total sample into two groups. The criteria for inclusion in the group of students with learning disabilities were adequate performance in Raven’s progressive matrices and a score of two standard deviations or less in a least one of the reading, writing and calculation tests. Tests used in a clinical environment to identify the existence of SLDs were employed for the screening; all tests used were standardized in Italian language.

First of all, to rule out intellectual disability, the intelligence quotient (IQ) was assessed using Raven’s Colored Progressive Matrices test [65]. In the whole sample, intellectual disability was excluded by reaching the 50th percentile corresponding to chronological age in Raven’s Colored Progressive Matrices test. We then administered reading, writing and calculation tests. Therefore, the following tests were administered: (a) MT-2 reading tests for secondary school [66]; (b) test 6 (dictation of words) and 7 (dictation of pseudo-words) of the battery DDE-2, a battery for the evaluation of dyslexia and developmental dysorthographia [67]; and (c) collective tests of the AC-MT 11-14, a test for the assessment of mathematical skills and problem solving [68].

In particular, the MT-2 reading tests for secondary school are one of the most commonly utilized instruments by specialists to obtain a functional evaluation of reading, particularly decoding and comprehension skills. The MT accuracy and speed test was used in this study. By reading aloud the provided materials, these tests determine the individual’s automatisms, such as the correctness of the decoding, the precision of the scanning, and the speed of reading the words of the written text. All students were given a maximum of four minutes to read the piece aloud without using their fingers to follow along. The reading times were timed, and any errors were recorded in order to compute raw and normalized accuracy and speed scores (time/syllable or syllable/time). To describe the typical amount of time required to read a syllable, the time/syllable rapidity indices, which are measured in hundredths of a second, were evaluated.

Instead, the DDE-2 battery is used to evaluate dyslexia and developmental dysorthography. It allows for assessing the level of competence in reading and writing in children attending from the 2nd class of primary school to the 3rd class of lower secondary school. The DDE-2 is one of the main tools available to specialists to assess the pupils’ competence in reading and writing. In particular, it aims to ascertain the state of decoding skills but not comprehension skills to analyze their characteristics, and if they were inadequately developed. The battery consists of eight tests: five for analyzing the reading processes and three for analyzing the writing process. In this study, we used the writing tests 6 and 7. The test 6 is a dictation test of words of different lengths and orthographic complexity that evaluates spelling efficiency. The test 7 is a dictation test of non-words of varying length and orthographic complexity which evaluates the grapheme-phoneme conversion process. The assessment was conducted in the following mode: first, we dictated 48 items (from four to eight characters), which corresponded to words; next, we dictated another 24 items (from five to nine characters), which corresponded to non-words. The computation of raw and standardized scores was highlighted for errors.

Finally, AC-MT 11-14 is a test for ascertaining the level of calculus learning (basic assessment) and detecting calculation difficulties. The AC-MT test is made up of three parts, administered to children in different ways and at different times. The test used for this research was the collective paper-pencil test which includes five tests: Carry out the operations, Judgment of numbers, Transformation into figures, and Sorting of series from minor to major and from major to minor. This material was delivered simultaneously to all the components of the class or group of children, arranged as in-school checks with separate desks.

The tests were scored in accordance with the guidelines in the reference guides. All students with z scores lower than 1.5 in one or more school subjects were given the specific learning disability. This phase of screening revealed that 26 students had a single SLD, 28 had a mixed disorder, and 44 did not have a learning disability. Following screening, each student answered a questionnaire about their perceptions of social integration and peer victimization (see next sections). To prevent feeling judged, the students answered the questionnaire in an anonymous manner. For each participant, an alphanumeric code was nevertheless produced. This process was essential in determining a link between the surveys and the work conducted during the screening phase.

### 2.2. Measures

#### 2.2.1. Perceived Social Integration

Perceived social integration was measured by adapting Carron’s Group Environmental Questionnaire [69,70] to the Italian school context. The original tool version evaluates group cohesion in sports teams and measures group dynamics from an individual and collective perspective [69]. The items investigate the perception of individual relationships to the group and invite reflection about the group as a whole. Additionally, the scale assesses both instrumental (such as collective performance and the group’s goals) and affective (such as group connections) variables in group dynamics. This study included only items that assess the relational characteristics from an individual perspective in order to capture students’ social judgments of their personal link with their classmates. Indeed, in the presence of learning difficulties, pupils’ goals are individualized rather than widely shared as a group. The selected and modified items were then used to assess how well students had integrated at school based on perceived connections with classmates. The items administered were the following: (1) “*To me, my class represents one of the most important groups to which I belong*”; (2) “*I enjoy spending time with my classmates*”; (3) “*If for any reason I had to change class, I would miss my classmates*”; (4) “*I feel similar to many of my classmates*”. Students responded to the statements through a nine-point Likert scale (1 = Strongly Disagree and 9 = Strongly Agree). Cronbach’s alpha was 0.78.

#### 2.2.2. Peer Victimization

The *Florence Bullying and Victimization Scale* [58,71] was used to measure peer victimization. Only the ten items that assessed victimization were used in this study. Before asking the participants to answer the questions, a definition of the phenomenon was provided as described by Olweus [72]. This was deemed necessary by the author to avoid measuring the many several constructs related to the phenomenon, such as violence or derision, but lacking important bullying characteristics such as the imbalance of power, intentionality and repetition over time [58]. Students had answered to indicate how often in the last two or three months they were bullied in terms of physical (example item: “*I was beaten*”), verbal (example item: “*I was called ugly names*”), and indirect bullying (example item: “*My classmates ignored me*”). A 5-step Likert scale was used to collect the responses. Response options included: never (1), 1 or 2 times (2), 3 or 4 times per month (3), once a week (4), and several times a week (5). Cronbach’s alpha for this scale was 0.84.

## 3. Data Analysis and Results

The data collected were analyzed using SPSS Statistics—27 software [73]. To test our first two hypotheses, we conducted two analyses of covariance (ANCOVAs) to examine differences between groups (students with learning disabilities and students without learning disabilities) in victimization and perceived social integration, taking into account the gender differences. Hayes’s PROCESS macro [74]—PROCESS Model 4—was used to test our third hypothesis. In particular, we tested a simple mediation model to understand whether, based on what was suggested by the literature examined, a higher frequency of victimization among students with learning disabilities, compared to peers who do not have the disorder, could be explained by a lower perceived social integration.

Before proceeding with the analysis, we considered the preliminary treatment of the data [64]. The victimization scores were found to violate some of the assumptions and were transformed using the square root method [75]. Then, all study variables were considered standardized, and correlations assessed (Table 1). The results highlighted a significant negative correlation between a specific learning disorder’s presence (vs. absence) and perceived social integration (r = −0.21, *p* = 0.036). The correlations between the presence (vs. absence) of a specific learning disorder and victimization were non-significant (r = −0.11, *p* = 0.281). As to perceived social integration, we found a significant and negative correlation with victimization (r = −0.34, *p* < 0.01).

An analysis of covariance (ANCOVA) was conducted to test our first hypothesis. In the model, the grouping variable (students with SLD and students without SLD) was inserted as an independent variable, gender as a covariate, and victimization as a dependent variable. In contrast to our first hypothesis, the results revealed non-significant differences in victimization levels between groups (Table 2), both as regards the presence (vs. absence) of a learning disability (F_(1, 95)_ = 1.147, *p* = 0.29) and gender (F_(1, 95)_ = 0.186, *p* = 0.68).

Regarding the differences between groups in the levels of perceived social integration (Table 2), we conducted a further analysis of covariance (ANCOVA). The data showed the presence of statistically significant differences between students with learning disabilities and students without learning disabilities (F_(1, 96)_ = 4.463, *p* = 0.037, η^2^ = 0.05). The former reported lower levels of social integration (M = 6.31, SD = 2.22) than the latter (M = 7.12, SD = 1.35). The second hypothesis is therefore confirmed.

Interestingly, taking into consideration the different levels of perceived social integration based on the severity of the disorder, ANOVA analysis has highlighted the presence of statistically significant differences (F_(2, 95)_ = 3.34, *p* = 0.04, η^2^ = 0.07). In particular, post-hoc comparisons (Tukey test; Table 3) revealed that students with a mixed disorder reported lower levels of perceived social integration (M = 5.96, SD = 2.50) than both their peers with only one disorder (M = 6.70, SD = 1.85) and without a disorder (M = 7.13, SD = 1.35). No statistically significant differences in perceived social integration scores among students with only one disorder and students without learning disabilities have been found.

To test our third hypothesis, a mediation analysis was conducted (see Figure 1) using macro PROCESS [74]. In PROCESSs Model 4 the level of perceived social integration (M) was set as mediator in the relationship between the presence (vs. absence) of a specific learning disorder (X) and victimization (Y). The results of the analysis revealed the presence of a significant and negative impact of the presence of learning disabilities on perceived social integration (B = −0.42; SE = 0.20; 95% CI [−0.821, −0.029]; *p* = 0.036). As to the effect of perceived social integration on the criterion variable (victimization), the effect was significant and negative (B = −0.38; SE = 0.097; 95% CI [−0.567, −0.182]; *p* = < 0.001). The direct effect of the predictor on the criterion variable was non-significant (B = −0.38; SE = 0.19; 95% CI [−0.765, 0.006]; *p* = 0.053) but a significant and positive effect of total mediation (B = 0.16; bootstrapped SE = 0.093; 95% CI [0.011, 0.364]) has been found (F_(2, 95)_ = 8.141; R^2^ = 0.15; *p* = 0.001). Our third hypothesis can therefore be considered confirmed.

## 4. Discussion

Bullying is widespread in schools worldwide, negatively impacting students’ and communities’ well-being [76]. Studies conducted over the last 40 years revealed that many factors expose children and adolescents to the risk of being victimized by their classmates. Notably, children and adolescents with disabilities are especially vulnerable to bullying [77,78]. The literature on these issues has shown that learning difficulties experienced by children and adolescents with SLDs may also have social consequences. Musetti and colleagues [30] demonstrated in the Italian setting that learning difficulties are not only reflected in the academic context but can harm students’ social interactions and socio-affective dynamics. It appears that children and adolescents with SLDs have a more difficult time distinguishing social cues and rules than their typically developing peers. This lack of social skill acquisition hinders the development of appropriate and functional interactions in the educational setting. Other processes may explain the greater prevalence of victimization among students with SLDs. Indeed, students with disabilities may be perceived as “different” by their peers depending on specific characteristics [45]. Several studies have indicated that diversity can increase the likelihood of being a victim of peer hostility (physical, verbal, or psychological) (e.g., [55]). Indeed, school bullying may be exacerbated by stereotypes, prejudices, and the social stigma associated with diversity [79,80,81]. Social ties, categorization processes, and group dynamics can also explain aggressive behavior toward members of minority groups [79,80,81].

The bioecological model is critical for understanding the influence of social environments (school, home, peer group, community, and society) on the factors that make children and adolescents vulnerable to bullying [53]. Indeed, despite individual risk factors based on personal characteristics (from personality traits to internalizing or externalizing disorders, up to gender, sexual orientation, physical or learning disabilities), relational dynamics and social processes play a crucial role in school bullying. Consequently, being a vulnerable child does not automatically imply being bullied: the likelihood of being bullied by one’s peers also depends on the qualities of the social environments within which children and adolescents are placed. Decades of research have allowed us to identify supportive school environments as variables promoting well-being and psychosocial adaptability [82]. Although adolescents with SLDs typically report negative social experiences at school, research suggests that a sense of school belonging may help these students with their psychosocial problems. Indeed, belonging to a peer network can have a substantial impact on pupils’ social and academic development (e.g., [31]). The relational issues that typically accompany students with learning disabilities may thus help to explain the frequency of victimization [83].

Based on the research reviewed, our study assumed that relational difficulties, in addition to learning difficulties, may be predictive of victimization experiences. Thus, the primary purpose of our study was to assess victimization among adolescents with specific learning difficulties. We wanted to study the phenomenon’s mechanisms and processes from a psychosocial standpoint. To accomplish this, we compared the levels of victimization among students with SLDs and typically developing students. We also included perceived social integration with peers as a variable capable of explaining victimization. Despite the study’s shortcomings, addressed below, our findings indicated that a learning disability did not affect victimization levels. Instead, our research suggested that having a learning disability interferes with peer interactions and social integration within the peer group. This finding is consistent with empirical findings that students with learning disabilities have poor social skills, preventing them from connecting constructively with their peers [30,31,40,84,85]. Therefore, the study allows us to identify perceived social integration as a risk factor for the victimization of students with learning disabilities. Indeed, perceived social integration has emerged as a mediator variable in the direct connection between the presence/absence of a SLD and victimization. Students with SLDs report being bullied to a greater extent than their typically developing peers when they do not perceive the presence of meaningful relationships with their classmates. This result is consistent with research indicating that individuals strongly desire to form and sustain positive and lasting social interactions with others [86]. Indeed, the frustration of the psychological need to belong to self-relevant social groups appears to be related to lower levels of social well-being and psychological adaptation [87]. At the same time, the study indicates that school climate can be an important protective factor in integrating diversity and reducing aggressive behavior. According to the research, a warm and supportive school climate is associated with lower bullying rates [88] and higher social competence [51]. In fact, schools with a positive and inclusive climate have been found to foster stronger peer relationships and play a protective role in cases of victimization [30].

Despite the encouraging findings, our study has several limitations. First, the sample is too small, despite being relatively homogeneous. This evidence did not allow us to analyze the model considering the differences between students with a single disorder or a mixed disorder and their peers without an SLD. However, a post-hoc Monte Carlo power simulation [89] found that the present study has good power (>0.80) to test the mediation hypothesis in a sample of ninety-eight children and one mediator. Furthermore, our study was cross-sectional, which provided a description of the phenomenon but restricted the ability to draw causal inferences between the variables considered. Additionally, the measure used to assess perceived social integration has not yet been validated. Furthermore, the association of learning disabilities with other neurodevelopmental disorders, such as attention deficit hyperactivity disorder (ADHD), was not taken into account. Finally, only victimization was considered in the study. SLD children and adolescents, on the other hand, appear to be both bullies and victims, according to studies [40,42,45].

Despite its many shortcomings, one of the study’s strengths is that it emphasizes social dynamics as risk and protective variables for the well-being of students with learning disabilities. These factors are relevant and emerge as tools that educators might employ to promote pupils’ healthy growth. Being situated in a welcoming school setting where all students, including those with learning disabilities, can feel socially comfortable benefits their well-being and learning processes. In this situation, teachers play a key role. Several studies have demonstrated that teachers function as an “invisible hand” [90] in school settings, supporting inclusion processes and managing a variety of issues within group dynamics [91,92,93]. As a result of our investigation, we can reflect on how crucial it is for professional development to strengthen teachers’ knowledge, abilities, and capacities in order to make them aware of the social dynamics involved in the teaching-learning process.

From a clinical standpoint, our research can yield several implications, notably for diagnostic procedures. The findings emphasize the need for early SLD diagnosis. Early identification of the disorder can help teachers and the educational community create a schooling experience appropriate for the students’ capabilities and needs. According to our findings, attention should be paid to the learning processes and the integration and inclusion of students with learning disabilities within the class group. Students’ social skills and classroom climate, in addition their cognitive and academic learning characteristics, should be assessed to develop a successful intervention strategy.

In conclusion, future research on the psychological well-being of students with learning difficulties should look at the impact of peer relationships and group dynamics, which are prevalent in the school setting. It would be very important to look at how peer relationships and social status affect bullying and being bullied by using samples with a wider range of ages and types of disorders. Future research should also take comorbid disorders with SLDs into account. In light of the importance that social and relational contexts assume for students’ adaptation, it would also be interesting to investigate whether the presence of a supportive environment can moderate the relationship between the existence of a learning disability and the perception of integration into the class group. According to numerous studies, feeling like a part of a community entails feeling connected, supported, and included by your group. Numerous suggestions for strengthening anti-bullying treatments, particularly for adolescents who exhibit a variety of vulnerabilities, could come from this line of research. Additionally, enhancing the school environment would benefit the entire academic community. Positive social ties between students from various backgrounds can improve all children and teenagers’ ability to act as responsible citizens. The school is, in fact, an institution responsible not only for the diffusion of knowledge but also for the training of young people, the future global citizens.

## Figures and Tables

**Table 1:** Correlations.

	M	DS	1.	2.	3.
1. SLD			-		
2. Perceived social integration	6.68	1.92	−0.213 *	-	
3. Peer Victimization	1.51	.63	−0.110	−0.335 **	-

*Note*. SLD (Specific Learning Disabilities): student with learning disabilities (*n* = 44) and students without learning disabilities (*n* = 54). * *p* < 0.05; ** *p* < 0.01.

**Table 2:** Analyses of covariance (ANCOVAs): differences in mean scores in peer victimization and perceived social integration according to the presence/absence of Specific Learning Disabilities and gender.

	Grouping Variable	M	DS	N	F	Sign.	n^2^
Victimization	Students without SLD	1.58	0.68	44			
	Students with SLD	1.45	0.59	54			
	Total	1.51	0.63	98	1.147	0.287	0.01
	Female	1.47	0.58	39			
	Male	1,53	0.67	59			
	Total	1.51	0.63	98	0.186	0.667	0.00
Perceived Social Integration	Students without SLD	7.12	1.35	44			
	Students with SLD	6.31	2.22	54			
	Total	6.68	1.92	98	4.463	0.037	0.05
	Female	6.33	2.19	39			
	Male	6.90	1.69	59			
	Total	6.68	1.92	98	2.040	0.157	0.02

*Note.* SLD (Specific Learning Disabilities.

**Table 3:** Result of Tukey’s post-hoc test for perceived social integration between groups (students without SLD, students with only one disorder, students with mixed disorders).

DV: Perceived Social Integration						
					CI (95%)	
(I) SLD	(J) SLD	Difference average (I-J)	SE	Sign.	Lower Limit	Upper Limit
Students without SLD	Students with only one disorder	0.43	0.46	0.62	−0. 67	1.53
	Students with mixed disorders	1.17 *	0.45	0.03	0.09	2.25
Students with only one disorder	Students without SLD	−0.43	0.46	0.62	−1.53	0.67
	Students with mixed disorders	0.74	0.51	0.32	−0.48	1.95
Students with mixed disorders	Students without SLD	−1.17 *	0.45	0.03	−2.25	−0.09
	Students with only one disorder	−0.74	0.51	0.32	−1.95	0.48

*Note.* SLD = Specific Learning Disabilities; * *p* < 0.05; students without SLD (*n* = 44), student with only one disorder (*n* = 26), students with mixed disorders (*n* = 28).

## Data Availability

The data presented in this study are available on request from the corresponding author. The data are not publicly available since data shared are not in accordance with consent provided by participants on the use of data.
